# Biomonitoring of Phthalates, Bisphenols and Parabens in Children: Exposure, Predictors and Risk Assessment

**DOI:** 10.3390/ijerph18178909

**Published:** 2021-08-24

**Authors:** Pablo Dualde, Nuria León, Yovana Sanchis, Francisca Corpas-Burgos, Sandra F. Fernández, Cristina S. Hernández, Guillermo Saez, Erika Pérez-Zafra, Antonio Mora-Herranz, Olga Pardo, Clara Coscollà, Antonio López, Vicent Yusà, on behalf of the BIOVAL Task Force

**Affiliations:** 1 Foundation for the Promotion of Health and Biomedical Research of the Valencian Region, FISABIO-Public Health, Av. Catalunya, 21, 46020 Valencia, Spain; dualde_pab@gva.es (P.D.); corpas_fra@gva.es (F.C.-B.); fernandez_sanfer@gva.es (S.F.F.); acrishern@gmail.com (C.S.H.); pardo_olg@gva.es (O.P.); coscolla_cla@gva.es (C.C.); lopez_anttob@gva.es (A.L.); 2 Public Health Laboratory of Valencia, Av. Cataluña, 21, 46020 Valencia, Spain; leon_nur@gva.es (N.L.); sanchis_yov@gva.es (Y.S.); 3 Conselleria Sanitat, Universitary Hospital Doctor Peset, Av. Gaspar Aguilar, 90, 46017 Valencia, Spain; Guillermo.Saez@uv.es (G.S.); eripeza@gmail.com (E.P.-Z.); tonimora65@gmail.com (A.M.-H.); 4 Analytical Chemistry Department, University of Valencia, Edifici Jeroni Muñoz, Dr. Moliner 50, 46100 Burjassot, Spain

**Keywords:** biomonitoring, phthalates, bisphenols, parabens, urine, risk assessment

## Abstract

Exposure to emerging contaminants, such as phthalates, bisphenols and parabens in children has been associated with possible neurodevelopment and endocrine alterations. In the present study, the biomonitoring of biomarkers in children (5–12 years old) from the Valencia Region (Spain) have been implemented using urines from the BIOVAL program. More than 75% of the children studied (*n* = 562) were internally exposed (>LOQ) to bisphenols and parabens, and the whole population assessed (*n* = 557) were exposed to at least one phthalate. The geometric means (GM) of the concentrations of bisphenol A, methyl paraben and propyl paraben were 0.9, 1.4 and 0.39 ng/mL, respectively. Regarding phthalates, monoethyl phthalate GM was 55.0 ng/mL and diethyl hexyl phthalate (as the sum of five metabolites) GM was 60.6 ng/mL. Despite the studied population being widely exposed, the detection frequencies and concentrations were in general lower than in previous studies involving children in Spain and in other countries in recent years. Furthermore, the risk assessment study concluded that the internal exposure to phthalates, bisphenols and parabens is lower than the guidance values established, and, therefore, a health risk derived from the exposure to these compounds in the studied population is not expected.

## 1. Introduction

The exposure to chemicals which interact with the organism is a public health issue, since it can cause alterations in the neurodevelopment or in the endocrine system [1]. During the last decades, there has been an increase in the number of studies which assess the health alterations derived from the exposure to emerging contaminants, such as bisphenols, phthalates and parabens.

Bisphenols are mainly used in food contact materials composed of polycarbonate plastics, in epoxy resins used in the internal coating of cans and in thermal papers [2]. Regarding children, the presence of bisphenols in toys can also be a source of exposure [3]. The most widely used and produced bisphenol is the bisphenol A (BPA), however, recent restrictions in its use have motivated the increase of production of BPA analogues, such as bisphenols F (BPF) and S (BPS) [4]. The exposure to bisphenols has been associated with possible alterations in the endocrine system [5]. Using toxicologic data, a temporal TDI (t-TDI) of 4 μg/Kg body weight (bw) per day has been established for the oral exposure to BPA [6].

Phthalates are plasticizers used in many applications. Long-chain phthalates, such as di-2-ethylhexyl phthalate (DEHP) and diisononyl phthalate (DiNP) are mainly used in polyvinyl chloride (PVC) materials, such as food contact materials, flooring, clothing or toys. On the other hand, short-chain phthalates, such as dimethyl phthalate (DMP), diethyl phthalate (DEP), benzylbutyl phthalate (BzBP) and diisobutyl phthalate (DiBP), are also used in personal care products, paints and in enteric-coated tablets [7]. The use of phthalates in the European Union (EU) is regulated in food contact materials [8], toys and other objects intended for child-use which can be used in the mouth [9], cosmetics [10], medicines [11] and medical devices [12]. Several studies have assessed the toxicity of phthalates in animals, and have concluded that they have negative effects on reproduction and development. Furthermore, they are considered possible endocrine disruptors in humans [13]. Regarding neurodevelopment, prenatal exposure to phthalates has been associated with negative cognitive development of children (e.g., lower intelligence quotient, attention deficit, hyperactivity and lower social communication) [14].

Parabens are chemicals used as preservatives [15]. The most widely produced parabens are methyl paraben (MP), ethyl paraben (EP), propyl paraben (PP) and butyl paraben (BP) [16]. The EU allows and regulates the use of parabens in food [17], medicines [18] and cosmetics [19]. Given the wide use of these compounds, researchers have detected levels in water [20] and other matrices [15]. Some studies with animals have correlated exposure to parabens with health effects, such as alterations in the estrogenic activity during gestation and childhood [21,22,23]. The EFSA has established an acceptable daily intake (ADI) for the sum of MP and EP of 0–10 mg/kg bw per day [24].

These contaminants can be absorbed by ingestion, inhalation or dermal absorption. After entering the body, they are metabolized by phase I and/or phase II metabolites and are excreted in urine in a few hours. Bisphenols are excreted in urine mainly as conjugates [25]. Parabens have been found in urine unaltered, hydrolyzed or oxidized; furthermore, they can be conjugated with glucuronide or sulfate [26]. Regarding phthalates, they are hydrolyzed by their monoesters, and subsequently, some of them are oxidized. In urine, they can be found as free metabolites or conjugated with glucuronic acid [27].

The ongoing Human Biomonitoring for Europe (HBM4EU) project attempts to provide policy makers with comparable data on human internal exposure to chemicals and mixtures of chemicals at EU level. This project has included emerging chemicals, such as phthalates and bisphenols, in the list of priority substances which are going to be assessed within the project [28]. Unlike other countries such as Germany [29], in Spain, there is not yet a national biomonitoring program which would evaluate the exposure to phthalates, bisphenols and parabens. The BIOVAL program attempts to evaluate the exposure to these substances in children from Valencia (Spain).

The objectives of the present study are: (i) to assess the urinary levels of biomarkers of bisphenols, phthalates and parabens in the infant population of the Valencia Region (Spain); (ii) study the risk assessment in the population; and (iii) assess the predictors of these levels using food consumption and socio-demographic questionnaires.

## 2. Materials and Methods

### 2.1. Study Area and Population

The present study is included in the BIOVAL program developed in the Valencia Region. The study design was described by Perez et al., [30]. The program is focused on the biomonitoring of food contaminants in children from the Valencia Region. During 2016, a total of 666 children (5 to 12 years old) from 25 primary schools of Alicante, Castellón and Valencia provinces were recruited. The parents/guardians of all the participants signed an informed consent document. The program was approved by the Ethical Committees of the Valencian Research Centre for Public Health (FISABIO) of the Valencian Government (Directorate-General of Public Health).

### 2.2. Sample and Data Collection

First-morning void (FMV) urine samples were collected during 2016 by the parents/guardians of the participants using gloves. Samples were collected in polypropylene containers with polyethylene caps. Previously to the sample collection, the container batches were tested following migration studies, in order to check the absence of phthalates or bisphenols. Samples were kept refrigerated and transported (<24 h) in a cooler with ice to the Biobank for Biomedical Research and Public Health of the Valencian Community (IBSP-CV Biobank) (PT13/0010/0064) integrated in the Spanish National Biobank Network and the Valencian Biobanking Network, where they were divided into aliquots and stored at −80 °C. For bisphenols, parabens and phthalate metabolite determination the aliquots were preserved in glass containers.

The participants’ parents/guardians completed self-administered food consumption and socio-demographic questionnaires. The characteristics of the studied population, including the frequency of food consumption by groups in the population (grams/month), are shown in Table 1 and Table S1.

### 2.3. Determination of Urinary Biomarkers

Since some participants provided low sample volumes and some of them presented with unusual levels of urinary creatinine (see the last part of Section 2.3), only 562 samples were analyzed for the determination of bisphenols and parabens, and 557 samples were analyzed for the determination of phthalate metabolites. The urinary biomarkers of bisphenols, parabens and phthalates determined in the present study are shown in Table S2. The determination of bisphenols and parabens in urine was implemented following the method described in Sanchis et al. [31]. Firstly, the enzymatic hydrolysis was implemented by adding 200 µL of 1M ammonium acetate buffer (pH 5.0) and 10 μL of β-glucuronidase/arylsulfatase to 500 µL of sample. After incubation (37 °C, 90 min), the samples were centrifuged and the supernatant was transferred into an injection vial. The determination was implemented by high performance liquid chromatography coupled with tandem mass spectrometry, using atmospheric pressure chemical ionization in negative mode. Regarding the phthalate metabolites determination, the sample preparation was described in Dualde et al. [32]. Briefly, 500 μL of urine were diluted with 660 μL of ultrapure water, 200 μL of 1M ammonium acetate buffer and 10 μL of enzyme β-glucuronidase (from E. coli K12). Then, the samples were incubated (90 min, 37 °C) and centrifuged. In total, 200 μL of the supernatant were transferred to the injection vial, and 10 μL were injected in the liquid chromatography coupled with a tandem mass spectrometry (LC-MS/MS) system, using electrospray ionization in negative mode.

Regarding quality assurance and quality control, samples were analyzed under quality system protocols following the ISO/IEC/EN 17,025 requirements. The limit of quantification (LOQ) was defined as the lowest level at which an analyte can be determined with acceptable recovery and precision. The procedural LOQ for each compound is presented in Table 2. Quality control samples to check the performance of the method were used in each batch, including reagent blanks, matrix blanks and spiked blank samples according to the SANTE/11813/2017 guideline [33]. The following performance criteria were used: recoveries within 70–120% and repeatability RSD ≤ 20%. Regarding linearity, R^2^ > 0.98 was obtained. Furthermore, the laboratory participated in the G-EQUAS inter-comparison program, round 62, in 2018, for the available urinary phthalate metabolites (MEHP, MECPP, MEHHP, MEOHP, MBzP, MnBP and MiBP) at two concentration levels, and in the ICI-EQUAS inter-comparison program, round 03, in 2019, for the available urinary phthalate metabolites (MEP, MEHP, MECPP, MEHHP, MEOHP, MBzP, MnBP, MiBP, MCHP and MOP) at two concentration levels, to guarantee the reliability of the results. The laboratory successfully fulfilled the requirements for all the phthalate metabolites evaluated in both programs.

The creatinine determination was implemented at the University Hospital Doctor Peset (Valencia, Spain) using Jaffé’s reaction. The samples with urinary creatinine outside of the normal range (0.3–3 g/L) [34] were excluded (*n* = 7).

### 2.4. Statistical Analysis

Statistical analysis was performed using R software (version 3.5.2). Urinary biomarker levels below limit of quantification (LOQ) were estimated following the maximum likelihood estimation method described in [35]. This method assumes that the data are distributed according to a certain parametric distribution, and estimates the parameters of this distribution by maximizing the probability of obtaining the observed sample. A log-normal distribution was assumed for the biomarker levels in urine.

First, a descriptive analysis of all the study variables was performed. Qualitative variables were described by absolute frequencies and percentages. Quantitative variables were summarized by their median and range. Additionally, biomarkers levels in urine were summarized by calculating the minimum, 25th, 50th, 75th and 95th percentiles, arithmetic and geometric means, maximum and standard deviation (Table 2).

Multiple logistic regression models were built to study associations between the presence of bisphenols and parabens in urine, and the socio-demographic and dietetic characteristics of the participants. The presence of phenols in urine (levels above the limit of detection) was considered a dependent variable in the models. The independent variables were sex, BMI, parent’s country of birth, maximum level of education of one of the parents, employment situation, MED-DQI index and food consumption. In order to directly compare the magnitude of regression coefficients, the numerical explanatory variables were centered and divided by twice their standard deviation, as proposed by Gelman [36]. The models were built following a forward variable selection procedure based on the Akaike Information Criterion (AIC) [37]. The quality of the fit of models was assessed using the Hosmer–Lemeshow test, exploring the model residuals (verifying that there are no observations with large residues) and calculating the area under the receiver operator characteristic (ROC) curve.

Multiple robust linear regression models were built to assess the relationship between the concentration of each phthalate in urine, and socio-demographic and dietary variables. The logarithmic transformation of phthalate levels in urine was considered to obtain the normality of the response variables (see Figure S1). Robust regression was considered as an alternative to ordinary least squares estimation methods (OLS), given the presence of some outlier values in phthalate levels. In order to directly compare the magnitude of regression coefficients, the numerical explanatory variables were centered and divided by twice their standard deviation, as proposed by Gelman [36]. The models were built following a backward variable selection procedure based on the robust final prediction error [38]. Confidence intervals at 95% significance were calculated for the model coefficients, to indicate the precision and uncertainty of the sample statistical estimates.

### 2.5. Risk Assessment

The urinary levels of BPA and phthalate metabolites were compared with their reference HBMs and/or biomonitoring equivalent (BEs) values (Table S3 [39]).

In the present study, a conservative scenario was chosen. If there was more than one BE for each biomarker, the lowest BE was selected as the guidance value. The hazard quotient (HQ) was calculated as the quotient between P95 of the biomarker levels in the population with the above-mentioned BEs or HBM-I values. See Equation (1):

HQ = P95/Guidance value(1)

The HQs were used to evaluate the health risk of the child population. If a biomarker concentration is below the guidance value (HQ < 1), a health risk is not expected. Nevertheless, a health risk cannot be discarded if the HQ > 1.

Regarding paraben levels, there were no guidance values for these compounds in urine. Therefore, the estimated daily intake (EDI) was calculated in order to implement the risk assessment by using reverse dosimetry [40]. See Equation (2):

EDI (mg/kg bw day) = (C (mg/L) V_urine_ (L/day))/F BW(kg)) (2)

where C is the paraben concentration, V_urine_ is the urinary volume excreted per day, F is the compound’s urinary excretion factor and BW is the body weight. Since a conservative scenario was followed, C was the 95th percentile (P95) of the paraben levels. The total volume of urine for children (6–12 years old) was 0.66 L per day according to Remer et al. [41]. There was no data available for the urinary excretion factor of parabens, and consequently a conservative F of 0.25 was selected. The median body weight of the participants was 32 kg (Table 1). The risk assessment was estimated by comparing the EDI calculated with the acceptable daily intake (ADI) of 0–10 mg/kg bw/day established by EFSA [24]. Consequently, the risk assessment for the sum of MP and EP was implemented using the HQ according to Equation (3):

HQ = (ΣEDI calculated)/(ADI)(3)

## 3. Results

### 3.1. Biomarker Urinary Levels

The levels of urinary biomarkers of bisphenols, parabens and phthalates in the population studied are shown in Table 2. In total, 75% of children had detectable levels of at least one bisphenol, with BPA being the most widely detected (DF 63%), and BPS levels were detected in almost 30% of the samples while BPF presented a DF of 11%. Regarding parabens, 88% of the children had detectable levels of one of the four parabens studied, with MP and PP being the ones which shown the highest DF (62.3 and 59.6%, respectively). In the case of phthalates, all participants had quantifiable levels of at least one biomarker. Nine out of the fourteen phthalate metabolites studied presented DFs > 77%. The rest of phthalate metabolites were only detected in a few samples (DF < 5%).

Regarding the measured concentrations, in case of bisphenols, BPA showed the highest geometric mean (GM) (0.9 ng/mL), while the levels of BPF and BPS were much lower (GMs < 0.2 ng/mL). The paraben which showed the highest levels was MP (GM 1.4 ng/mL), followed by PP (GM = 0.39 ng/mL). BP showed the lowest concentrations. In the case of phthalates, the metabolite which presented the highest concentrations was MEP, the biomarker of DEP. However, DEHP, as the sum of five metabolites, was the phthalate which presented the highest levels of metabolites in urine (GM = 60.6 ng/mL).

### 3.2. Determinants

The determinants of the biomarkers which presented DFs > 40% were assessed. Table 3 shows the multiple logistic regression models for the presence of BPA, MP, EP and PP in urine. The consumption of eggs, canned fish and processed fish was negatively associated with the levels of BPA in urine, however, the consumption of white fish and drinks were positively associated with BPA concentrations.

Regarding parabens, participants who had foreign fathers showed lower levels of MP and PP. Furthermore, female children presented with significatively higher levels of PP, and a fewer adhesion to the Mediterranean diet quality index was associated with higher EP levels.

Table 4 shows the robust multiple linear regression model for the logarithmically adjusted levels of phthalate metabolites. Regarding the frequency of food consumption, a higher consumption of molluscs was associated with higher levels of a BzBP metabolite (MBzP) and two DEHP metabolites (2cx-MMHP and MEHP). The consumption of small blue fish was positively associated with MEP levels, however, it was negatively associated with MEOHP levels. Furthermore, the consumption of eggs was negatively associated with the levels of MnBP, and a higher ingestion of drinks was associated with higher levels of MEP and a low adhesion to the Mediterranean diet quality index was associated with higher MEP levels. Regarding socio-demographic variables, the children with foreign parents presented higher levels of MnBP, the children with both parents unemployed had lower levels of MBzP, and the girls presented lower levels of MEHP. Since only 11% of the participants had foreign parents and only 6% of children had both parents unemployed, these associations could be inconsistent.

### 3.3. Risk Assessment

**Figure 1:**
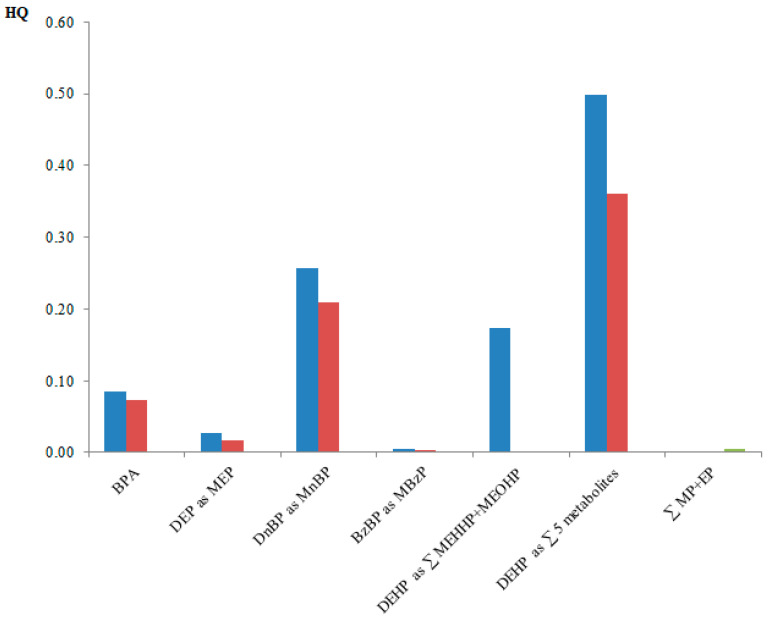
HQs calculated in the present study. Blue columns: HQs calculated using ng/mL; red columns: HQs calculated using µg/g _creatinine_; green columns: for parabens only, HQ calculated using EDI and ADI.

Figure 1 shows the HQs for all compounds assessed using the P95 values. None of the contaminants studied had HQs higher than one, therefore a health risk is not expected. The phthalates DEHP (as the sum of five metabolites in ng/mL) and DnBP (as MnBP in ng/mL) showed the highest HQs (0.50 and 0.26, respectively). The lowest HQs were for ∑MP + EP (in mg/kg bw day) and BzBP (as MBzP in ng/mL) (0.05 and 0.047, respectively).

## 4. Discussion

The assessment of children’s internal exposure to bisphenols, parabens and phthalates through urine analysis revealed the wide exposure to these compounds in the Valencia Region (Spain). In total, 75% of children had been exposed to at least one bisphenol, 88% to at least one paraben and 100% to at least one phthalate. Previously, other studies worldwide have assessed the exposure of children to these compounds. Table 5 shows the levels of these three groups of substances in the urine of children in international studies since 2016, and with a number of participants higher than 200.

BPA was the most frequently detected bisphenol in all studies, followed by BPS and BPF, except in the study developed in Hokkaido (Japan) whereby BPF was slightly more frequently detected than BPS. BPA also showed the highest concentrations in all studies. In comparison with the Valencian children, the study developed in Canada showed similar concentrations (GMs 0.90 and 0.97 ng/mL, for the present study and the Canadian study, respectively). On the contrary, the two studies developed in China (in Jiangsu and in Guangzhou) and the one developed in USA showed higher levels. However, the studies developed in Hokkaido and South Korea showed lower levels than in the present study.

Regarding parabens, in all studies, the MP was the compound most frequently detected and showed the highest concentrations, while BP showed the lowest detection frequencies and concentrations. In comparison with the present study, the rest of the studies showed higher levels of DF for all parabens except BP, which in the present study had the highest DF (21.5%).

With reference to phthalates, the DFs were high (80–100%) in most of the studies, and DEHP metabolites levels were similar or higher in studies developed in Iran, Italy, South Korea, China, Europe and Czech Republic. Only the studies developed in Canada, USA and Germany showed lower levels of DEHP metabolites than in the present study. Regarding biomarkers of short-chain phthalates, all studies showed lower levels of MEP (metabolite of DEP), however, the levels of DnBP, DiBP and BzBP biomarkers (MnBP, MiBP and MBzP, respectively) were similar or higher in the rest of studies in comparison with the present study, and only the study developed in China showed lower levels of MBzP.

Table S4 shows the levels of bisphenols, parabens and phthalates urinary biomarkers in studies involving Children in Spain. As can be seen, BPA was previously studied in children from Sabadell, Granada, Madrid and Añover de Tajo [58,59,60]. The BPA DFs were >90% in all the previous studies, however in our study, the DF% was lower (63%). Furthermore, the GMs in previous studies were in a range from 1.87 to 5.8 µg/g creatinine, while in our study the GM was much lower (0.92 µg/g creatinine). As far as we know, the levels of BPF and BPS have not been previously studied in Spanish children.

Regarding parabens, Casas et al. [61] studied the urinary levels of MP, EP, PP and BP in children from Granada, and despite their LOD being equal or lower than in our study, the DF% were higher (80–100%) than in our study (21–62%). Furthermore, the levels in the Granada children were also much higher, with medians ranging from 1.2 to 150 ng/mL, while in the Valencian children the median range was <0.2 to 2.4 ng/mL. On the other hand, both in Valencian and Granada children the parabens with the highest DFs and concentration were MP and PP.

Urinary phthalate metabolites were studied in children from Madrid, Añover de Tajo and Granada [60,61]. The DFs of phthalate metabolites were slightly higher in previous studies, and the concentrations were much higher, especially in Añover de Tajo, with GMs ranging from 7.5 to 259.8 µg/g creatinine, while in Valencian children the GMs ranged from 3.0 to 55.9 µg/g creatinine. The present study was the largest study implemented in Spanish children, with more than 500 samples, while the other studies had 19 to 172 participants.

The fact that the Valencian study showed lower levels of DFs could be related to the year of sampling, since in previous studies the samples were collected between 2005 and 2012, while in the Valencian study the samples were collected in 2016. The reduction in the use of these products and the substitution with analogues, such as BPF and BPS in the case of BPA, or DINCH in the case of phthalates, could explain this reduction.

With reference to the predictor study, we have not found an explanation to the negative association between BPA levels in urine and the consumption of eggs, canned fish and processed fish. It should be expected that a higher consumption of canned food would be related with a higher exposure to BPA, since BPA is present in the epoxy resins used to internally coat the cans [2]. On the other hand, BPA levels were positively associated with consumption of white fish and drinks. Gys et al. [62] studied the levels of bisphenols in Flemish adolescents and also found a relation between BPA levels in urine and consumption of local fish. Martinez et al. [63] observed that the reduction of canned drinks during pregnancy in women from Spain was related with lower levels of BPA in urine. Regarding parabens, girls had significatively higher levels of PP than boys. This fact could be related to a higher exposition to parabens through the use of personal care products. However, we cannot confirm this hypothesis, since we do not have data of cosmetic use. A lower adherence to a Mediterranean diet was associated with higher levels of EP and MEP (metabolite of DEP). However, Ax et al. [64] studied the correlation between Mediterranean diet and phthalate levels in blood, and did not find significant correlations. EP can be used as a food additive as a preservative, therefore, it is possible that people with a higher adherence to a Mediterranean diet consume less food containing additives. Children with higher consumption of molluscs showed higher levels of metabolites of BzBP and DEHP, and children with higher consumption of small white fish showed higher levels of the biomarker of DEP. Sánchez-Ávila et al., [65] detected levels of DEHP in mussels from three out of five coastal and estuarine sites studied in the Cantabrian Sea (Spain). Furthermore, Castro-Jiménez & Ratola [66] detected levels of DEHP in both wild and commercial mussels, levels of BzBP in wild mussels and levels of DEP in fish from the Mediterranean Sea. Furthermore, boys showed higher urinary levels of MEHP (DEHP metabolite) than girls from the present study, however, we have not found an explanation for this association.

The risk assessment study showed that the internal exposure of the Valencian children to BPA, MP, EP and DEP, DnBP, BzBP and DINP is of low concern, using the guidance values as a reference. However, emerging data show that exposure to low levels of these contaminants can be associated with adverse outcomes [67]. Furthermore, some oxidized DINP metabolites as mono(hydroxyl-isononyl) phthalate and mono(oxo-isononyl) phthalate [68] should be studied in future studies, in order to assess the internal exposure to DINP. Furthermore, some oxidized metabolites of BP have been described and detected in urine, and could improve the biomonitoring of this paraben in the future [26].

## 5. Conclusions

This is the largest biomonitoring study of urinary biomarkers of phthalates, bisphenols and parabens in Spanish children. Despite the children being widely exposed to these chemicals, the detection frequencies and levels were lower than in other studies involving children in other regions of Spain, and were lower, in general, than recent studies developed in other countries. The risk assessment study concluded that the internal exposure to these emerging chemicals is of low concern for the Valencian children. The future study of other oxidized metabolites of phthalates and parabens will improve the assessment of the internal exposure of these compounds.

## Figures and Tables

**Table 1:** Studied population characteristics.

	*n* (%) (*n* = 562)
Province	
Alicante	203 (36.12%)
Castellón	122 (21.71%)
Valencia	237 (42.17%)
Sex	
Male	282 (50.18%)
Female	280 (49.82%)
Size (cm)	135 (100–170) ^a^
*Missing data*	17 (3.02%)
Weight (Kg)	32 (16–72) ^a^
*Missing data*	11 (1.96%)
BMI	17.11 (11.65–45.45) ^a^
*Missing data*	18 (3.20%)
Age	8 (5–12) ^a^
Child’s country of birth	
Spain	552 (98.75%)
Foreign	7 (1.25%)
*Missing data*	3 (0.53%)
Parents country of birth	
Both Spain	488 (89.38%)
Some foreign	58 (10.62%)
*Missing data*	16 (2.85%)
Years of child’s residence in the Valencia Region	8 (1–12) ^a^
*Missing data*	11 (1.96%)
Maximum level of education of one of the parents	
Without studies or primary	56 (9.96%)
Secondary	169 (30.07%)
Superior	337 (59.96%)
Employment situation	
Neither parents work	33 (5.87%)
Some of the parents work	529 (94.13%)
Primary sector work	
None	536 (95.37%)
Someone	26 (4.63%)
Secondary sector work	
None	341 (60.68%)
Someone	221 (39.32%)
Tertiary sector work	
None	100 (17.79%)
Someone	462 (82.21%)
Index MED-DQI	4 (0–10) ^a^
Good (score ≤ 4)	340 (60.5%)
Medium-good (5 ≤ score ≤ 7)	196 (34.88%)
Medium-poor (8 ≤ score ≤ 10)	26 (4.63%)

^a^ Values expressed as median (minimum–maximum). BMI (body mass index). MED-DQI (Mediterranean diet quality index).

**Table 2:** Levels of contaminant biomarkers in urine samples.

Analyte	*n*	DF (%)	LOQ ng/mL	Minimum ng/mL (µg/g Creat)	P25 ng/mL (µg/g Creat)	GM ng/mL (µg/g Creat)	Median ng/mL (µg/g Creat)	P75 ng/mL (µg/g Creat)	P95 ng/mL (µg/g Creat)	Maximum ng/mL (µg/g Creat)	Std dev ng/mL (µg/g Creat)
BPA	562	63.3	0.2	<LOQ	<LOQ	0.90 (0.92)	1.6 (1.8)	10.4 (9.9)	85.2 (95.2)	6246.2 (6277.6)	294.4 (299.3)
BPF	562	11.6	0.2	<LOQ	<LOQ	<LOQ	<LOQ	<LOQ	3.2 (2.6)	2571.5 (4056.0)	155.5 (218.5)
BPS	562	28.6	0.2	<LOQ	<LOQ	<LOQ	<LOQ	0.30 (0.31)	6.8 (6.1)	153.5 (96.5)	10.0 (7.8)
∑Bisphenols	562	75.6	-	-	-	2.3 (2.3)	3.3 (3.6)	13.4 (13.7)	154.2 (154.0)	6246.2 (6277.6)	337.7 (377.6)
MP	562	62.3	0.2	<LOQ	<LOQ	1.4 (1.4)	2.4 (2.5)	61.7 (60.6)	541.4 (574.4)	23210.0 (27598.1)	1100.0 (1253.5)
EP	562	48.4	0.2	<LOQ	<LOQ	<LOQ	<LOQ	2.0 (2.2)	18.0 (21.0)	910.7 (1081.6)	51.6 (58.6)
PP	562	59.6	0.2	<LOQ	<LOQ	0.39 (0.40)	0.40 (0.41)	2.3 (2.4)	61.3 (61.3)	378.7 (527.0)	43.7 (44.5)
BP	562	21.5	0.2	<LOQ	<LOQ	<LOQ	<LOQ	<LOQ	7.3 (9.1)	475.2 (568.5)	56.6 (70.2)
∑Parabens	562	88.1	-	-	-	9.4 (9.5)	8.7 (8.5)	80.5 (80.5)	833.7 (881.6)	23498.0 (27940.6)	1124.4 (1282.3)
MEP	557	99.8	2	<LOQ	26.9 (26.7)	55.0 (55.9)	51.1 (53.2)	100.6 (105.1)	498.0 (405.4)	8273.0 (13,343.5)	438.5 (612.0)
MiBP	557	98.6	2	<LOQ	9.7 (10.7)	18.4 (18.7)	18.4 (17.6)	31.6 (32.0)	83.2 (85.4)	1039.2 (659.8)	56.0 (44.2)
MnBP	557	99.6	0.5	<LOQ	8.2 (8.7)	14.0 (14.2)	13.8 (13.6)	23.7 (21.6)	51.5 (58.6)	309.4 (325.1)	30.9 (31.8)
MBzP	557	88.2	1	<LOQ	1.5 (1.5)	3.0 (3.0)	2.8 (2.9)	5.6 (5.4)	17.8 (19.5)	110.7 (89.4)	11.0 (9.6)
2cxMMHP	557	77.9	2	<LOQ	2.2 (2.4)	4.0 (4.1)	4.0 (4.1)	7.1 (6.8)	18.5 (19.5)	93.3 (171.7)	9.0 (11.1)
MEOHP	555	100	0.5	0.7 (1.1)	5.2 (5.4)	9.1 (9.2)	9.3 (9.2)	15.9 (15.0)	37.9 (34.2)	267.2 (352.4)	17.3 (23.0)
MEHHP	557	98.7	2	<LOQ	7.0 (7.4)	11.9 (12.1)	11.9 (11.4)	18.5 (18.2)	53.1 (48.3)	292.7 (628.1)	23.0 (33.5)
MECPP	557	100	1	1.8 (2.1)	15.5 (15.6)	27.0 (27.5)	27.9 (29.1)	50.6 (45.9)	101.0 (90.5)	480.7 (890.3)	39.2 (50.7)
MEHP	557	84.6	1	<LOQ	1.8 (1.7)	3.6 (3.6)	3.7 (3.8)	7.7 (7.5)	24.0 (25.2)	99.7 (117.2)	9.4 (11.3)
DEHP(∑MEHHP + MEOHP)	555	100	-	-	12.9 (13.7)	21.6 (21.9)	22.4 (29.1)	34.6 (33.1)	86.9 (78.6)	459.3 (980.5)	37.6 (54.2)
DEHP (∑5metabolites) ^a^	555	100	-	-	35.1 (37.5)	60.6 (61.6)	62.1 (61.8)	101.8 (97.6)	214.5 (198.8)	1044.8 (2159.7)	84.6 (117.3)
∑Phthalates	555	100	-	-	113.9 (115.4)	185.9 (188.9)	176.2 (176.8)	289.0 (285.7)	703.2 (673.4)	8356.8 (13,478.7)	463.8 (635.7)

*n* (number of samples); MP: Methyk paraben; EP: Ethyl Paraben; PP: Propyl paraben; BP: Butyl paraben; MEP: Mono-ethyl phthalate; MiBP: Mono-isobutyl phthalate; MnBP: Mono-n-butyl phthalate; MBzP: Mono-benzyl phthalate; 2cxMMHP: Mono[2-(carboxymethyl)hexyl] phthalate; MEOHP: Mono-(2-ethyl-5-oxohexyl) phthalate; MEHHP: Mono-(2-ethyl-5-hydroxyhexyl) phthalate; MECPP: Mono-(2-ethyl-5-carboxypentyl) phthalate; MEHP: Mono-(2-ethyl-5-hydroxyhexyl) phthalate; DF (detection frequency); LOQ (limit of quantification); GM (geometric mean); P25 (25th percentile); P75 (75th percentile); P95 (95th percentile); Std dev (standard deviation); creat (creatinine). ^a^ Sum of MEHP, MEOHP, MECPP, MEHHP and 2cx-MMHP. Other metabolite LOQs (ng/mL): MCPP (2); MiNP (0.5); MCHP (0.5); MMP (1); MOP (0.5). MiNP was not detected in any sample; MCHP was detected in 1 sample (0.84 ng/mL); MMP was detected in 3 samples (8.1–268.4 ng/mL); MOP was detected in 4 samples (0.50–0.51ng/mL); and MCPP was detected in 17 samples (2.5–47.2 ng/mL). Limit of detection (LOD) was calculated as 1/3 LOQ.

**Table 3:** Results of the multiple logistic regression model for presence of BPA, MP, EP and PP in urine (levels ≥ LOQ).

Variable	Standardized Coefficients (95% CI)	Standard Error	*p*-Value	Odd Ratio (95% CI)
**BPA**				
Intercept	0.5621 (0.3819–0.7457)	0.0927	<0.0001 *	-
Canned fish	−0.611 (−1.0895–−0.1863)	0.2332	0.0088 *	0.5428 (0.3364–0.83)
Processed fish	−0.3781 (−0.7703–−0.0167)	0.1897	0.0463 *	0.6851 (0.4629–0.9835)
White fish	0.3996 (0.0228–0.7886)	0.1949	0.0404 *	1.4912 (1.023–2.2002)
MED-DQI index	−0.2953 (−0.6718–0.0778)	0.1909	0.1219	0.7443 (0.5108–1.0809)
Drinks	0.4916 (0.054–1.0248)	0.2462	0.0459 *	1.6349 (1.0555–2.7865)
Eggs	−0.2042 (−0.3918–−0.0203)	0.0944	0.0306 *	0.8153 (0.6758–0.9799)
**MP**				
Intercept	1.0693 (0.4807–1.7133)	0.3121	0.0006 *	-
Eggs	0.2288 (−0.1292–0.5917)	0.1834	0.2122	1.2571 (0.8788–1.807)
Drinks	−0.1802 (−0.5406–0.1711)	0.1784	0.3125	0.8351 (0.5824–1.1866)
Maximum level of education of one of the parents: secondary	−0.3742 (−1.0886–0.2996)	0.3522	0.2880	0.6879 (0.3367–1.3493)
Maximum level of education of one of the parents: superior	−0.6319 (−1.3087–−0.0036)	0.3308	0.0561	0.5316 (0.2702–0.9964)
Parents country of birth: some foreign	−0.5752 (−1.1375–−0.0133)	0.2856	0.0440 *	0.5626 (0.3206–0.9868)
**EP**				
Intercept	−0.7776 (−1.5772–−0.0472)	0.3857	0.0438 *	-
Fishing products	0.1886 (−0.2073–0.5905)	0.2027	0.3522	1.2076 (0.8128–1.805)
Bivalve molluscs	−0.1624 (−0.6232–0.2355)	0.2142	0.4485	0.8501 (0.5362–1.2655)
Employment situation: some of the parents work	0.7389 (−0.0129–1.5565)	0.396	0.0620	2.0936 (0.9871–4.742)
MED-DQI index	0.4045 (0.0521–0.7627)	0.181	0.0254 *	1.4986 (1.0535–2.1442)
**PP**				
Intercept	0.2386 (−0.011–0.4905)	0.1278	0.0619	-
Sex: female	0.4281 (0.079–0.7796)	0.1786	0.0165 *	1.5343 (1.0822–2.1805)
Parents country of birth: some foreign	−0.746 (−1.325–−0.1781)	0.2913	0.0104 *	0.4742 (0.2658–0.8368)
Nuts	−0.2255 (−0.5896–0.1213)	0.1787	0.2069	0.7981 (0.5545–1.1289)
White fish	0.2829 (−0.0703–0.6443)	0.1819	0.1198	1.327 (0.9321–1.9047)
Creatinine	0.2978 (−0.059–0.6639)	0.184	0.1055	1.3469 (0.9427–1.9423)

* *p*-value ≤ 0.05.

**Table 4:** Results of the robust multiple linear regression model for the logarithmically adjusted levels of phthalate metabolites.

Variable	Standardized Coefficients (95% CI)	Standard Error	*p*-Value	
**MnBP**				
Intercept	2.7426 (2.4768–3.0084)	0.1356	<0.0001 *	
Employment situation: some of the parents work	−0.1904 (−0.4626–0.0818)	0.1389	0.1709	
Fishing products	−0.0825 (−0.2316–0.0666)	0.0761	0.2786	
Nuts	−0.056 (−0.1902–0.0782)	0.0685	0.4142	
Legumes, potatoes and cereals	0.1008 (−0.0323–0.2339)	0.0679	0.1386	
Miscellany	−0.147 (−0.2761–−0.0179)	0.0659	0.0261 *	
Molluscs	0.1121 (−0.032–0.2562)	0.0735	0.1278	
Creatinine	0.5694 (0.4399–0.6989)	0.0660	<0.0001 *	
Parents country of birth: foreign	0.2621 (0.0499–0.4743)	0.1083	0.0158 *	
**MEP**				
Intercept	4.3685 (4.0006–4.7364)	0.1877	<0.0001 *	
Employment situation: some of the parents work	−0.4829 (−0.8617–−0.1041)	0.1933	0.0128 *	
Vegetables and fruits	−0.1712 (−0.355–0.0126)	0.0938	0.0685	
Drinks	0.2734 (0.0893–0.4575)	0.0939	0.0038 *	
White fish	−0.1469 (−0.3335–0.0397)	0.0952	0.1234	
Small blue fish	0.2173 (0.0325–0.4021)	0.0943	0.0215 *	
Creatinine	0.2868 (0.1104–0.4632)	0.0900	0.0015 *	
Index MED-DQI	0.1944 (0.0116–0.3772)	0.0932	0.0376 *	
**MBzP**				
Intercept	0.6615 (0.2973–1.0257)	0.1858	0.0004 *	
Sex: female	−0.1634 (−0.328–0.0012)	0.084	0.0521	
Employment situation: some of the parents work	0.4865 (0.1252–0.8478)	0.1843	0.0086 *	
Drinks	−0.1339 (−0.3112–0.0434)	0.0905	0.1393	
Molluscs	0.1739 (0.0106–0.3372)	0.0833	0.0374 *	
Creatinine	0.5207 (0.3569–0.6845)	0.0836	<0.0001 *	
**2-cx-MMHP**				
Intercept	1.3302 (1.2469–1.4135)	0.0425	<0.0001 *	
Small blue fish	−0.1193 (−0.2777–0.0391)	0.0808	0.1404	
Molluscs	0.2479 (0.0815–0.4143)	0.0849	0.0037 *	
Creatinine	0.6442 (0.4878–0.8006)	0.0798	<0.0001 *	
Parents country of birth: foreign	0.2467 (−0.0081–0.5015)	0.1300	0.0583	
**MEOHP**				
Intercept	2.167 (2.0976–2.2364)	0.0354	<0.0001 *	
Small blue fish	−0.1423 (−0.2841–−5e−04)	0.0724	0.0498 *	
Creatinine	0.6157 (0.4763–0.7551)	0.0711	<0.0001 *	
**MEHHP**				
Intercept	2.3829 (2.3098–2.456)	0.0373	<0.0001 *	
Small blue fish	−0.106 (−0.2453–0.0333)	0.0711	0.1365	
Creatinine	0.6033 (0.464–0.7426)	0.071	<0.0001 *	
Parents country of birth: foreign	0.1711 (−0.0516–0.3938)	0.1136	0.1326	
**MECPP**				
Intercept	3.2709 (3.1991–3.3427)	0.0366	<0.0001 *	
Canned molluscs	−0.142 (−0.3147–0.0307)	0.0881	0.1078	
Canned fish	0.0925 (−0.1038–0.2888)	0.1001	0.3562	
Creatinine	0.6402 (0.4964–0.784)	0.0734	<0.0001 *	
**MEHP**				
Intercept	1.7405 (1.2842–2.1968)	0.2328	<0.0001 *	
Sex: female	−0.2312 (−0.4412–−0.0212)	0.1072	0.0314 *	
Employment situation: some of the parents work	−0.3765 (−0.8282–0.0752)	0.2304	0.1029	
Fish	−0.2053 (−0.4223–0.0117)	0.1107	0.0643	
Molluscs	0.2233 (0.0086–0.438)	0.1095	0.0420 *	
Canned fish	0.191 (−0.0181–0.4001)	0.1067	0.0740	
Creatinine	0.6193 (0.4045–0.8341)	0.1096	<0.0001 *	
**MiBP**				
Intercept	3.22 (2.8943–3.5457)	0.1662	<0.0001 *	
Employment situation: some of the parents work	−0.3651 (−0.6971–−0.0331)	0.1694	0.0316 *	
Eggs	−0.1845 (−0.3372–−0.0318)	0.0779	0.0182 *	
Water	−0.1395 (−0.2913–0.0123)	0.0774	0.0722	
Creatinine	0.5634 (0.4112–0.7156)	0.0776	<0.0001 *	
Parents country of birth: foreign	0.211 (−0.0282–0.4502)	0.122	0.0845	

* *p*-value ≤ 0.05.

**Table 5:** Urinary levels of bisphenols, parabens and phthalates in children in large studies (*n* > 200) with samples collected since 2016.

Analyte	Country (City or Region)	Year SAMPLING	Age (Years)	Sample Size	LOQ (ng/mL)	DF%	GM ng/mL	Median ng/mL	P95 ng/mL	Reference
							(μg/g Creat)	(μg/g Creat)	(μg/g Creat)	
**BPA**	Canada	2016–2017	6–11	516	0.32 *	88.6	0.97	0.94	5.5	[42]
	China (Jiangsu)	2016–2017 (approx) **	7	412	0.01 *	99.3	4.66	2.41	287	[43]
	Japan (Hokkaido)	2012–2017	7	396	0.3	89	-	0.89	-	[44]
	USA	2013–2016	6–11	965	0.2	-	1.28	-	-	[45]
	South Korea	2017–2018	7–12	286	0.24 ***	85	0.7	0.53	4.9	[46]
	China (Guangzhou)	2014–2017	6–12	250	0.25 ***	87.2	1.7	2.24	14.4	[47]
	Spain (Valencia Region)	2016	6–11	562	0.2	63.3	0.90 (0.92)	1.6 (1.8)	85.2 (95.2)	Present study
**BPF**	Japan (Hokkaido)	2012–2017	7	396	0.02	83	-	0.07	-	[44]
	South Korea	2017–2018	7–12	286	0.19 ***	9.4	-	<0.19	0.54	[46]
	Spain (Valencia Region)	2016	6–11	562	0.2	11.6	<0.2	<0.2	3.2 (2.6)	Present study
**BPS**	Japan (Hokkaido)	2012–2017	7	396	0.04	78	-	0.11	-	[44]
	USA	2013–2016	6–11	965	0.1	-	0.36	-	-	[45]
	South Korea	2017–2018	7–12	286	0.13 ***	75.2	0.23	0.21	1.1	[46]
	Spain (Valencia Region)	2016	6–11	562	0.2	28.6	<0.2	<0.2	6.8 (6.1)	Present study
**MP**	Canada	2016–2017	6–11	540	1.3 *	88.4	7.5	4.9	-	[42]
	South Korea	2015–2017	6–11	839	-	-	-	18.1	-	[48]
	Germany	2014–2017	3–17	490	0.5	97	7.724	5.13	517	[49]
	Spain (Valencia Region)	2016	6–11	562	0.2	62.3	1.4 (1.4)	2.4 (2.5)	541.4 (574.4)	Present study
**EP**	Canada	2016–2017	6–11	540	0.9 *	26.3	-	<0.9	-	[42]
	South Korea	2015–2017	6–11	839	-	-	-	10.4	-	[48]
	Germany	2014–2017	3–17	516	0.5	69	0.943	0.73	12.0	[49]
	Spain (Valencia Region)	2016	6–11	562	0.2	48.4	<0.2	<0.2	18.0 (21.0)	Present study
**PP**	Canada	2016–2017	6–11	540	0.3 *	70.3	0.96	0.69	-	[42]
	South Korea	2015–2017	6–11	839	-	-	-	1.3	-	[48]
	Germany	2014–2017	3–17	516	0.5	31	0.563	<0.5	18.5	[49]
	Spain (Valencia Region)	2016	6–11	562	0.2	59.6	0.39 (0.40)	0.40 (0.41)	61.3 (61.3)	Present study
**BP**	Canada	2016–2017	6–11	540	0.3 *	7.2	-	<0.3	<0.3	[42]
	Germany	2014–2017	3–17	516	0.5	2	<0.5	<0.5	<0.5	[49]
	Spain (Valencia Region)	2016	6–11	562	0.2	21.5	<0.2	<0.2	7.3 (9.1)	Present study
**MEHP**	Iran (Isfahan)	2016	6–18	242	-	99.6	59.09	61.27	-	[50]
	Canada	2016–2017	6–11	534	0.11 *	99.9	1.4	1.4	5.8	[42]
	Europe ****	2013–2016	6–12	1260	0.15–1 *	96.8	-	(2.88)	-	[51]
	USA	2015–2016	6–11	415	0.8	-	1.42	1.30	5.9	[52]
	Czech Republic	2016	5 and 9	370	2	62.4	2.3	-	7.3	[53]
	Germany	2015–2017	6–10	736	0.5	86	1.4	1.5	6.8	[54]
	Italy	2015–2017	4–14	900	0.58	99.3	9.26	8.90	23.48	[55]
	China (Shenzhen)	2016–2017	6–8	1490	0.2	97	(6.69)	(7.30)	(33.3)	[56]
	Spain (Valencia Region)	2016	5–12	557	1	84.6	3.6 (3.6)	3.7 (3.8)	24.0 (25.2)	Present study
**MEOHP**	Iran (Isfahan)	2016	6–18	242	-	95.9	178.72	270.92	-	[50]
	Canada	2016–2017	6–11	537	0.17 *	100	7.0	7.5	31	[42]
	Europe ****	2013–2016	6–12	1300	0.12–0.5 *	99.9	-	(12.5)	-	[51]
	South Korea	2015–2017	6–11	839	-	-	-	19.7	-	[48]
	USA	2015–2016	6–11	415	0.2	-	5.97	6.1	24.5	[52]
	Czech Republic	2016	5 and 9	370	2	98.6	12.9	-	41.3	[53]
	Germany	2015–2017	6–10	736	0.2	100	9	9	32.1	[54]
	Italy	2015–2017	4–14	900	0.48	99.8	11.58	10.94	50.14	[55]
	China (Shenzhen)	2016–2017	6–8	1490	0.2	80	(6.54)	(12.8)	(53.2)	[56]
	Spain (Valencia Region)	2016	5–12	555	0.5	100.0	9.1 (9.2)	9.3 (9.2)	37.9 (34.2)	Present study
**2cxMMHP**	Canada	2016–2017	6–11	537	0.27 *	98.8	3.1	3.1	13	[42]
	China (Shenzhen)	2016–2017	6–8	1490	0.2	100	(7.96)	(7.59)	(36.5)	[56]
	Spain (Valencia Region)	2016	5–12	557	2	77.9	4.0 (4.1)	4.0 (4.1)	18.5 (19.5)	Present study
**MECPP**	Canada	2016–2017	6–11	535	0.28 *	100	13	13	52	[42]
	Europe ****	2013–2016	6–12	1300	-	99.9	-	(35.1)	-	[51]
	South Korea	2015–2017	6–11	839	-	-	-	44.9	-	[48]
	USA	2015–2016	6–11	415	0.4	-	14.6	14.9	60.3	[52]
	Germany	2015–2017	6–10	736	0.2	100	14.1	13.8	47.7	[54]
	China (Shenzhen)	2016–2017	6–8	1490	0.1	100	(19.8)	(20.3)	(78.0)	[56]
	Spain (Valencia Region)	2016	5–12	557	1	100.0	27.0 (27.5)	27.9 (29.1)	101.0 (90.5)	Present study
**MEHHP**	Iran (Isfahan)	2016	6–18	242	-	96.3	114.20	177.56	-	[50]
	Canada	2016–2017	6–11	537	0.22 *	100	9.7	9.9	44	[42]
	Europe ****	2013–2016	6–12	1298	0.12–0.5 *	99.8	-	(20.1)	-	[51]
	South Korea	2015–2017	6–11	839	-	-	-	30.2	-	[48]
	USA	2015–2016	6–11	415	0.4	-	8.81	9.0	39.0	[52]
	Czech Republic	2016	5 and 9	370	1.5	100	20.6	-	66.3	[53]
	Germany	2015–2017	6–10	736	0.2	100	12.7	12.9	43.6	[54]
	Italy	2015–2017	4–14	900	0.24	99.2	24.40	23.55	102.38	[55]
	China (Shenzhen)	2016–2017	6–8	1490	0.3	100	(23.1)	(22.2)	(85.7)	[56]
	Spain (Valencia Region)	2016	5–12	557	2	98.7	11.9 (12.1)	11.9 (11.4)	53.1 (48.3)	Present study
**MEP**	Canada	2016–2017	6–11	536	0.98 *	99.5	18	15	-	[42]
	Europe ****	2013–2016	6–12	1301	0.15–1 *	100	-	(33.5)	-	[51]
	USA	2015–2016	6–11	415	1.2	-	24.5	22.1	211	[52]
	Germany	2015–2017	6–10	736	0.5	100	21.7	19.8	165	[54]
	China (Shenzhen)	2016–2017	6–8	1490	0.3	98	(14.3)	(13.8)	(142)	[56]
	Spain (Valencia Region)	2016	5–12	557	2	99.8	55.0 (55.9)	51.1 (53.2)	498.0 (405.4)	Present study
**MnBP**	Iran (Isfahan)	2016	6–18	242	-	100	165.26	260.72	-	[50]
	Canada	2016–2017	6–11	536	0.6 *	100	20	19	84	[42]
	Europe ****	2013–2016	6–12	1301	0.15–1 *	100	-	(23.9)	-	[51]
	South Korea	2015–2017	6–11	839	-	-	-	45.0	-	[48]
	USA	2015–2016	6–11	415	0.4	-	14.4	15.4	47.2	[52]
	Czech Republic	2016	5 and 9	370	1.6	100	63.0	-	233.0	[53]
	Germany	2015–2017	6–10	736	1	100	22.9	22.5	80.2	[54]
	Thailand (Bangkok)	2016	2–18	221	-	88.6	(214.4)	(252)	-	[57]
	China (Shenzhen)	2016–2017	6–8	1490	0.1	99	(185)	(212)	(832)	[56]
	Spain (Valencia Region)	2016	5–12	557	0.5	99.6	14.0 (14.2)	13.8 (13.6)	51.5 (58.6)	Present study
**MiBP**	Canada	2016–2017	6–11	536	0.57 *	99.9	15	14	74	[42]
	Europe ****	2013–2016	6–12	1301	0.15–0.5 *	100	-	(41.8)	-	[51]
	USA	2015–2016	6–11	415	0.8	-	11.2	11.6	59.7	[52]
	Czech Republic	2016	5 and 9	370	2.7	100	44.1	-	233.6	[53]
	Germany	2015–2017	6–10	736	1	-	(27.1)	(24.7)	(97.9)	[54]
	China (Shenzhen)	2016–2017	6–8	1490	1.5	94	(32.1)	(36.8)	(176)	[56]
	Spain (Valencia Region)	2016	5–12	557	2	98.6	18.4 (18.7)	18.4 (17.6)	83.2 (85.4)	Present study
**MBzP**	Iran (Isfahan)	2016	6–18	242	-	100	173.17	240.77	-	[50]
	Canada	2016–2017	6–11	537	0.37 *	99.4	10	9.6	58	[42]
	Europe ****	2013–2016	6–12	1300	0.06–0.5 *	99.9	-	(5.0)	-	[51]
	South Korea	2015–2017	6–11	839	-	-	-	3.3	-	[48]
	USA	2015–2016	6–11	415	0.3	-	10.7	10.9	81.1	[52]
	Czech Republic	2016	5 and 9	370	1.9	71.6	3.7	-	32.9	[53]
	Germany	2015–2017	6–10	736	0.2	100	3.4	3.2	19	[54]
	China (Shenzhen)	2016–2017	6–8	1490	0.3	25	(0.44)	-	(2.75)	[56]
	Spain (Valencia Region)	2016	5–12	557	1	88.2	3.0 (3.0)	2.8 (2.9)	17.8 (19.5)	Present study

* LOD; ** sampling year not indicated, however, children were born between 2009 and 2010 and urine samples were collected at the age of 7 years old; *** method detection limit; **** six European countries (UK, France, Spain, Lithuania, Norway and Greece).

## Data Availability

The children data used to support the findings of this study are restricted in order to protect their privacy. Data are available from Vicent Yusà, yusa_vic@gva.es, for researchers who meet the criteria for access to confidential data.

## Supplementary Materials

The following are available online at www.mdpi.com/1660-4601/18/17/8909/s1. Figure S1. Histograms of Log-concentration of urinary phthalate metabolites; Table S1: Frequency of food consumption by groups in the population (grams/month); Table S2: Bisphenols parabens and phthalates and their urine biomarkers determined in the present study and their respective parents; Table S3: Biomonitoring equivalents (BE) and HBM I guidance values for BPA and phthalates in urine; Table S4: Urinary biomarker levels of bisphenols, parabens and phthalates in Spanish children.
