# Supplementary material for: Biomonitoring of Phthalates, Bisphenols and Parabens in Children: Exposure, Predictors and Risk Assessment
Source: Int J Environ Res Public Health. 2021 Aug 24;18(17):8909. doi: 10.3390/ijerph18178909 (PMC8431397; doi:10.3390/ijerph18178909)
Supplement: Supplementary file 1 [file ijerph-18-08909-s001.zip › ijerph-1293572-supplementary.pdf]

<u>*Supplementary Material*</u>

Biomonitoring of phthalates, bisphenols and parabens in children: exposure, predictors and risk assessment

Pablo Dualde [1*], Nuria León [2], Yovana Sanchis [2], Francisca Corpas-Burgos [1], Sandra F.Fernández [1], Cristina S.Hernández [1], Guillermo Saez [3], Erika Pérez-Zafra [3], Antonio Mora-Herranz [3], Olga Pardo [1], Clara Coscollà [1], Antonio López [1], Vicent Yusà [1,4,*], on behalf of the BIOVAL task force

[1] Foundation for the Promotion of Health and Biomedical Research of the Valencian Region, FISABIO-Public Health, Av. Catalunya, 21, 46020, Valencia, Spain.
[2] Public Health Laboratory of Valencia, Av. Cataluña, 21, 46020, Valencia, Spain.
[3] Universitary Hospital Doctor Peset, Av. Gaspar Aguilar, 90, 46017, Valencia, Spain.
[4] Analytical Chemistry Department, University of Valencia, Edifici Jeroni Muñoz, Dr. Moliner 50, 46100, Burjassot, Spain.

* Correspondence: yusa_vic@gva.es (V.Y.); dualde_pab@gva.es (P.D.)

**Table**	**Pages**
**Table S1.** Frequency of food consumption by groups in the population (grams/month).	1-2
**Table S2.** Mediterranean Diet Quality Index (Med-DQI) calculation	4
**Table S3.** Bisphenols parabens and phthalates and their urine biomarkers determined in the present study and their respective parents	5-8
**Table S4**. Biomonitoring equivalents (BE) and HBM I guidance values for BPA and phthalates in urine	9
**Table S5**. Urinary biomarker levels of bisphenols, parabens and phthalates in Spanish children	10-12
**Figure S1.** Histograms of Log-concentration of urinary phthalate metabolites	13

Table S1. Frequency of food consumption by groups in the population (grams/month).

Food	Median (minimum - maximum)
Dairy products	12309.5 (1621 - 47704.5)
Eggs	720 (30 - 1800)
Missing data [a]	3 (0.53 %)
Meat products	5017.5 (670 - 25275)
Missing data [a]	1 (0.18 %)
Fishing products	2820 (150 - 17430)
Vegetables and fruits	12496.5 (1020 - 48600)
Nuts	120 (15 - 2250)
Missing data [a]	7 (1.25 %)
Legumes, potatoes and cereals	8865 (1387.5 - 33325)
Oils and fats	547.5 (25 - 2271.3)
Missing data [a]	4 (0.71 %)
Pastries	1646 (101 - 12626)
Missing data [a]	1 (0.18 %)
Miscellany	1026.5 (60 - 20904)
Missing data [a]	2 (0.36%)
Water [b]	27000 (100 - 39000)
Missing data [a]	9 (1.6 %)
Drinks[b,c]	2188 (0 - 61731)
Fish	2120 (110 - 10350)
White fish	800 (100 - 6000)
Missing data [a]	3 (0.53 %)
Big blue fish	150 (75 - 3300)
Missing data [a]	17 (3.02 %)
Small blue fish	150 (75 - 1800)
Missing data [a]	12 (2.14 %)
Bivalve molluscs	100 (100 - 6000)
Missing data [a]	17 (3.02 %)
Crustaceans	50 (50 - 1200)

Missing data [a]	16 (2.85 %)
Cephalopods	240 (60 - 1440)
Missing data [a]	7 (1.25 %)
Canned molluscs	35 (35 - 2100)
Missing data [a]	18 (3.23%)
Canned fish	200 (50 - 7500)
Missing data [a]	13 (2.31 %)
Processed fish (surimi, …)	10 (10 - 440)
Missing data [a]	20 (3.56 %)

[a] Absolute frequency (percentage).

[b] mL/month

[c] Drinks group refers to: Water, soft drinks, juices, infusions and tiger nut milk,

Table S2. Mediterranean Diet Quality Index (Med-DQI) calculation.

**Score** (arbitrary units, "a. u.")	**SFA(Saturated fatty acids) (%energy)**	**Cholesterol (mg/day)**	**Meat (g/day)**	**Olive oil (mL/day)**	**Fish (g/day)**	**Legumes and cereals (g/day)**	**Fruits and vegetables (g/day)**
**0**	<10	<300	<25	>15	>60	>300	>700
**1**	10 - 13	300 - 400	25 - 125	5 - 15	30 - 60	100 - 300	400 - 700
**2**	>13	>400	>125	>5	>30	>100	>400

Calculation of Mediterranean Diet Quality Index (MED-DQI) was based on Gerber, 2006 **[1]**. Briefly, the percentage of saturated fatty acids (SFA) energy, cholesterol mg/day, meat g/day, olive oil mL/day, fish g/day, cereals and legumes g/day and fruits and vegetables g/day were calculated. Following the rules described in the Table above, diet was classified in: Good (1-4 arbitrary units, "a. u."), Half-good (5-7 a.u.), Half-poor (8-10 a.u.) and Poor (11-14 a.u.).

Table S3. Bisphenols parabens and phthalates and their urine biomarkers determined in the present study and their respective parents [2-4].

Contaminant (Acronym)	Biomarker (Acronym)	Structure	LoQ present study (ng/mL)
Bisphenol A (BPA)	Bisphenol A (BPA)	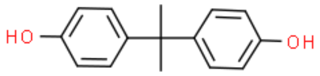	0.2
Bisphenol F (BPF)	Bisphenol F (BPF)	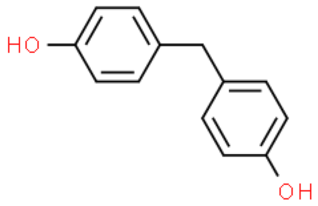	0.2
Bisphenol S (BPS)	Bisphenol S (BPS)	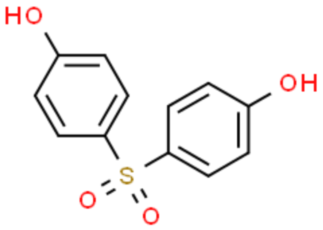	0.2
Methyl paraben (MP)	Methyl paraben (MP)	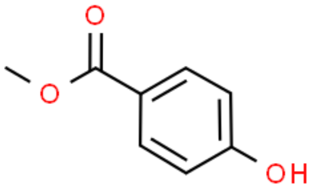	0.2

Ethyl paraben (EP)	Ethyl paraben (EP)	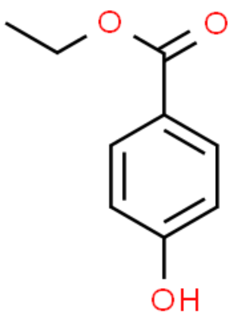	0.2
Propyl paraben (PP)	Propyl paraben (PP)	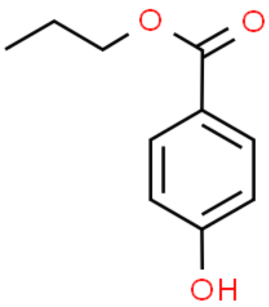	0.2
Butyl paraben (BP)	Butyl paraben (BP)	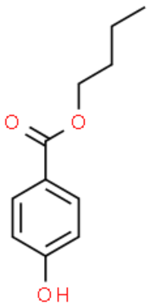	0.2
Di-2-ethylhexyl Phthalate (DEHP)	Mono-2-ethylhexyl phthalate (MEHP)	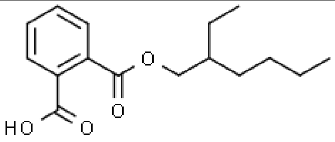	2
	Mono-(2-ethyl-5-oxohexyl) phthalate (MEOHP)	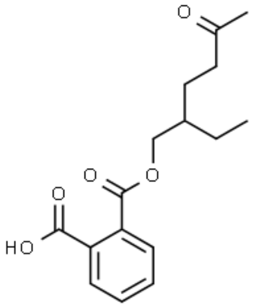	0.5
	Mono-(2-ethyl-5-carboxypentyl) phthalate (MECPP)	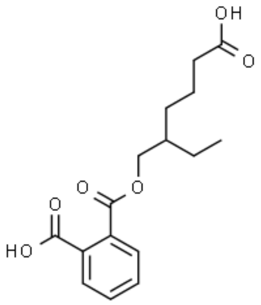	1

	Mono-(2-ethyl-5-hydroxyhexyl) phthalate (MEHHP)	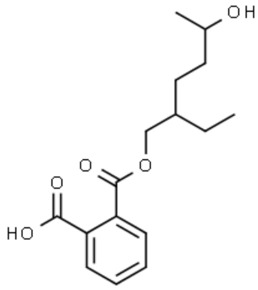	2
	Mono[2-(carboxymethyl)hexyl] phthalate (2cx-MMHP)	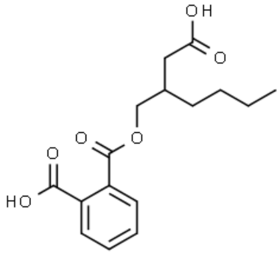	2
Di-isononyl Phthalate (DiNP)	Mono-isononyl phthalate (MiNP)	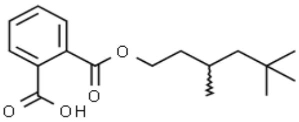	0.5
Diethyl Phthalate (DEP)	Mono-ethyl phthalate (MEP)	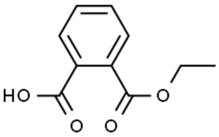	2
Di-n-butyl phthalate (DBP)	Mono-n-butyl phthalate (MnBP)	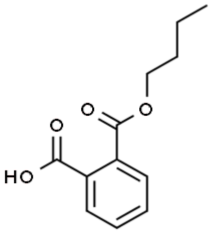	0.5
Di-isobutyl phthalate (DiBP)	Mono-isobutyl phthalate (MiBP)	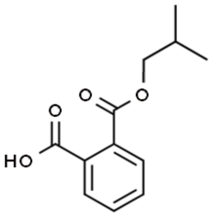	2
Benzylbutyl Phthalate (BzBP)	Mono-benzyl phthalate (MBzP)	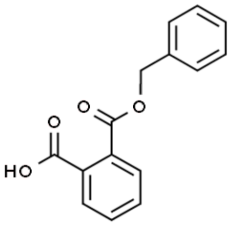	1
Dicyclohexyl Phthalate (DCP)	Mono-cyclohexyl phthalate (MCHP)	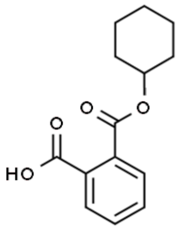	0.5

Di-(n-octyl) Phthalate(DOP)	Mono-(3-carboxypropyl) phthalate (MCPP)	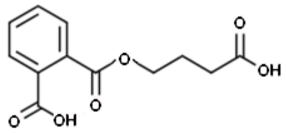	2
	Mono-n-octyl phthalate (MOP)	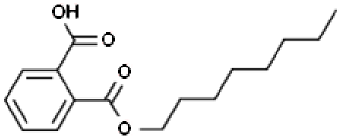	0.5
Dimethyl Phthalate (DMP)	Mono-methyl phthalate (MMP)	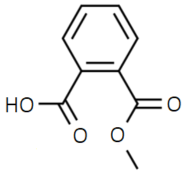	1

Table S4. Biomonitoring equivalents (BE) and HBM I guidance values for BPA and phthalates in urine.

**Analyte**	**BE**						**HBM I**	**Ref.**
	**Derived from Health Canada TDI**	**Derived from US EPA RfD**	**Derived from ATSDR chronic MRL**	**Derived from ATSDR Intermediate MRL**	**Derived from US CPSC**	**Derived from EFSA TDI**		
BPA ng/mL (µg/g creatinine)	1000* (1300)*	2000 (2600)	-	-	-	2000 (2600)	-	**[5]**
DEHP (as the sum of five metabolites: MEHP, MEHHP, MEOHP, MECPP, and 2cx-MMHP) ng/mL (µg/g creatinine)	1000 (1300)	430* (550)*	1300 (1700)	3200 (4100)	-	1100 (1400)	-	**[6]**
DEHP (as the sum of two metabolites: MEHHP and MEOHP) ng/mL	-	-	-	-	-	-	500[a]*	**[7]**
DEP (as MEP) ng/mL (µg/g creatinine)	-	18000* (23000)*	-	-	-	-	-	**[8]**
BzBP (as MBzP) ng/mL (µg/g creatinine)	31000 (40000)	3800* (4900)*	-	-	-	12000 (15000)	-	**[8]**
DnBP (as MnBP) ng/mL (µg/g creatinine)	1400 (1800)	2700 (3500)	-	-	-	200* (280)*	-	**[8]**
DiNP (as MiNP) ng/mL (µg/g creatinine)	170 (220)	-	-	-	72* (92)*	89 (110)	-	**[9]**

[a]For children aged 6-13 years

*Guidance values used in the present study

TDI (Tolerable Daily Intake); EPA (Environmental Protection Agency); ATSDR (Agency for Toxic Substances and Disease Registry); US CPSC (United States Consumer Product Safety Commission); EFSA (European Food Safety Agency)

Table S5. Urinary biomarker levels of bisphenols, parabens and phthalates in Spanish children.

Analyte	City or Region	Year sampling	Age (years)	Sample size	LoQ (ng/mL)	DF%	GM ng/mL (µg/g creat)	Median ng/mL (µg/g creat)	P95 ng/mL (µg/g creat)	Range ng/mL (µg/g creat)	Reference
BPA	Sabadell	2008-2010	4	130	0.1	100	3.1 (3.9)	3.1 (3.7)	12.3 (15.9)	0.5 (1.1) - 33.3 (36.2)	**[10]**
	Granada	2010-2012	9-11	172	0.2	100	5.1 (5.8)	5.03 (5.37)	23.2 (29.9)	0.25 (0.46) - 44.4 (63.7)	**[11]**
	Madrid (urban)	2011-2012	5-11	59	0.2	>90	(1.87)	-	(12.77)	(22.68)*	**[12]**
	Añover de Tajo (rural)	2011-2012	5-11	60	0.2	>90	(2.15)	-	(7.93)	(12.58)*	**[12]**
	Valencia Region	2016	5-12	562	0.2	63.3	0.90 (0.92)	1.6 (1.8)	85.2 (95.2)	<LoQ - 6246.2 (6277.6)	Present study
MP	Granada	2005-2006	4	30	1**	100	-	150	-	-	**[13]**
	Valencia Region	2016	5-12	562	0.2	62.3	1.4 (1.4)	2.4 (2.5)	541.4 (574.4)	<LoQ - 23210.0 (27598.1)	Present study
EP	Granada	2005-2006	4	30	0.2**	80	-	8.1	-	-	**[13]**
	Valencia Region	2016	5-12	562	0.2	48.4	<LoQ	<LoQ	18.0 (21.0)	<LoQ - 910.7 (1081.6)	Present study
PP	Granada	2005-2006	4	30	0.2**	100	-	21.5	-	-	**[13]**
	Valencia Region	2016	5-12	562	0.2	59.6	0.39 (0.40)	0.40 (0.41)	61.3 (61.3)	<LoQ - 378.7 (527.0)	Present study
BP	Granada	2005-2006	4	30	1**	86.3	-	1.2	-	-	**[13]**
	Valencia Region	2016	5-12	562	0.2	21.5	<LoQ	<LoQ	7.3 (9.1)	<LoQ - 475.2 (568.5)	Present study
MEHP	Madrid (urban)	2011-2012	5-11	59	0.5	≃100	(6.24)	-	(13.75)	(15.54)*	**[12]**
	Añover de Tajo (rural)	2011-2012	5-11	60	0.5	≃100	(7.50)	-	(19.36)	(43.85)*	**[12]**
	Granada	2005-2006	4	19	1.2**	100	-	6.2	-	-	**[13]**
	Valencia Region	2016	5-12	557	1	84.6	3.6 (3.6)	3.7 (3.8)	24.0 (25.2)	<LoQ - 99.7 (117.2)	Present study

**MEOHP**	Madrid (urban)	2011-2012	5-11	59	0.2	≃100	(21.23)	-	(49.36)	(70.90)*	**[12]**
	Añover de Tajo (rural)	2011-2012	5-11	60	0.2	≃100	(27.69)	-	(75.66)	(110.01)*	**[12]**
	Granada	2005-2006	4	19	0.7**	100	-	44.6	-	-	**[13]**
	Valencia Region	2016	5-12	555	0.5	100.0	9.1 (9.2)	9.3 (9.2)	37.9 (34.2)	0.7 (1.1) - 267.2 (352.4)	Present study
**MECPP**	Granada	2005-2006	4	19	0.6**	100	-	115.0	-	-	**[13]**
	Valencia Region	2016	5-12	557	1	100.0	27.0 (27.5)	27.9 (29.1)	101.0 (90.5)	1.8 (2.1)- 480.7 (890.3)	Present study
**MEHHP**	Madrid (urban)	2011-2012	5-11	59	0.2	≃100	(34.80)	-	(94.88)	(122.83)*	**[12]**
	Añover de Tajo (rural)	2011-2012	5-11	60	0.2	≃100	(42.21)	-	(110.94)	(151.86)*	**[12]**
	Granada	2005-2006	4	19	0.7**	100	-	57.4	-	-	**[13]**
	Valencia Region	2016	5-12	557	2	98.7	11.9 (12.1)	11.9 (11.4)	53.1 (48.3)	<LoQ - 292.7 (628.1)	Present study
**MEP**	Madrid (urban)	2011-2012	5-11	59	0.25	≃100	(151.6)	-	(1705)	(3425.7)*	**[12]**
	Añover de Tajo (rural)	2011-2012	5-11	60	0.25	≃100	(259.8)	-	(440.9)	(440.9)*	**[12]**
	Granada	2005-2006	4	19	0.8**	100	-	755	-	-	**[13]**
	Valencia Region	2016	5-12	557	2	99.8	55.0 (55.9)	51.1 (53.2)	498.0 (405.4)	<LoQ - 8273.0 (13343.5)	Present study
**MnBP**	Madrid (urban)	2011-2012	5-11	59	1	≃100	(46.92)	-	(150.9)	(237.1)*	**[12]**
	Añover de Tajo (rural)	2011-2012	5-11	60	1	≃100	(55.25)	-	(215.7)	(440.8)*	**[12]**
	Granada	2005-2006	4	19	0.6**	100	-	30.2	-	-	**[13]**
	Valencia Region	2016	5-12	557	0.5	99.6	14.0 (14.2)	13.8 (13.6)	51.5 (58.6)	<LoQ - 309.4 (325.1)	Present study
**MiBP**	Madrid (urban)	2011-2012	5-11	59	1	≃100	(60.73)	-	(172.8)	(187.1)*	**[12]**
	Añover de Tajo (rural)	2011-2012	5-11	60	1	≃100	(62.03)	-	(289.5)	(401.4)*	**[12]**
	Granada	2005-2006	4	19	0.3**	100	-	41.9	-	-	**[13]**
	Valencia Region	2016	5-12	557	2	98.6	18.4 (18.7)	18.4 (17.6)	83.2 (85.4)	<LoQ - 1039.2 (659.8)	Present study

**MBzP**	Madrid (urban)	2011-2012	5-11	59	0.2	≃100	(14.21)	-	(47.08)	(95.18)*	**[12]**
	Añover de Tajo (rural)	2011-2012	5-11	60	0.2	≃100	(13.60)	-	(52.46)	(135.5)*	**[12]**
	Granada	2005-2006	4	19	0.3**	100	-	33.0	-	-	**[13]**
	Valencia Region	2016	5-12	557	1	88.2	3.0 (3.0)	2.8 (2.9)	17.8 (19.5)	<LoQ - 110.7 (89.4)	Present study

* Maximum level

** Limit of detection (LoD)

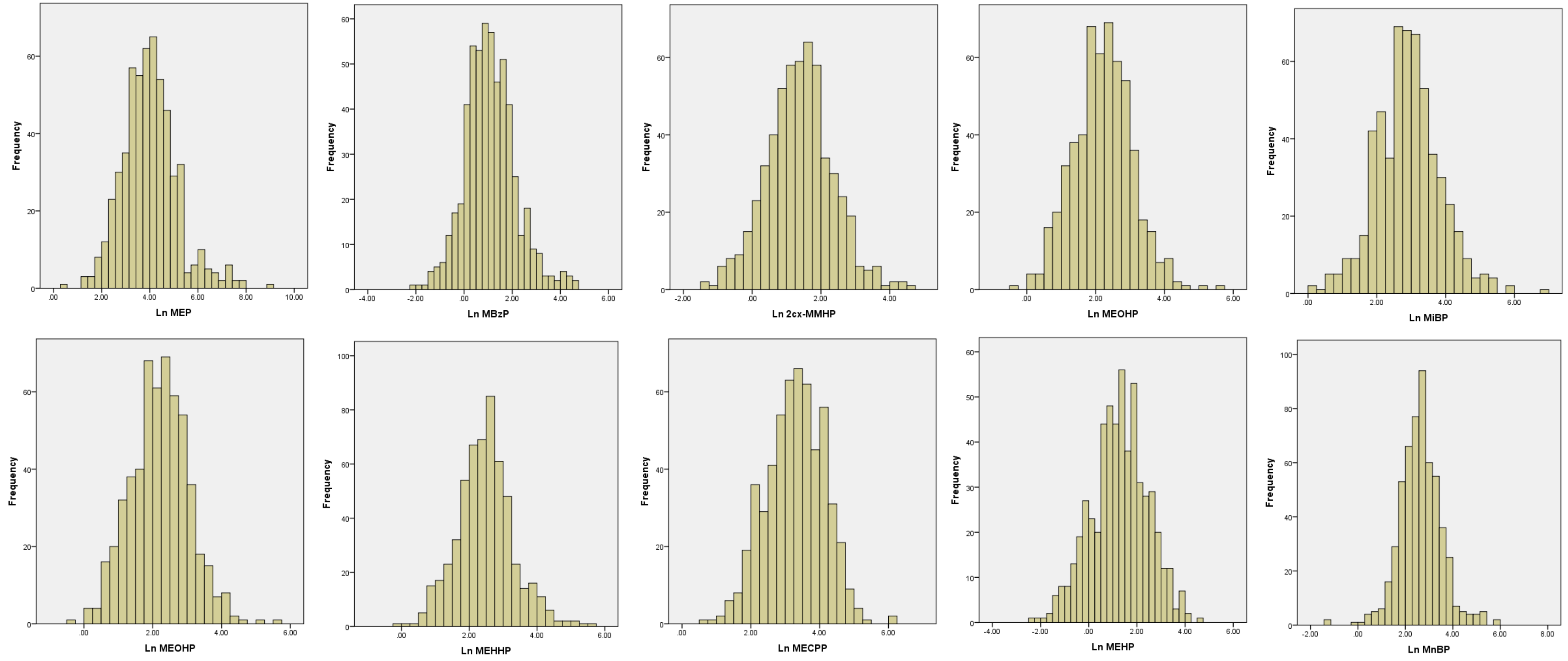

Figure S1. Histograms of Log-concentration of urinary phthalate metabolites.

References Supplementary Material

1. Gerber, M. Qualitative methods to evaluate Mediterranean diet in adults. Public Health Nutr. 2006, 9, 1a, 147-151. https://doi.org/10.1079/PHN2005937.
2. NHANES. 2009 Fourth National Report on Human Exposure to environmental chemicals. CDC. https://www.cdc.gov/exposurereport/pdf/fourthreport.pdf (Last visit: 12/04/2019).
3. Frederiksen, H.; Skakkebaek, N.E.; Andersson, A. Metabolism of Phthalates in Humans. Mol. Nutr. Food Res. 2007, *51*, 899-911. https://doi.org/10.1002/mnfr.200600243.
4. Rocha, B.A.; Moraes de Oliveira, A.R.; Barbosa Jr, F. A fast and simple air-assisted liquid-liquid microextraction procedure for the simultaneous determination of bisphenols, parabens, benzophenones, triclosan, and triclocarban in human urine by liquid chromatography-tandem mass spectrometry. Talanta 2018, 183, 94-101. https://doi.org/10.1016/j.talanta.2018.02.052.
5. Krishnan, K.; Gagne, M.; Nong, A.; Aylward, L.L.; Hays, S.M. Biomonitoring Equivalents for Bisphenol A (BPA). Regulatory Toxicology and Pharmacology 2010, 58, 18-24. https://doi.org/10.1016/j.yrtph.2010.06.005
6. Aylward, L.L.; Hays, S.M.; Gagne, M.; Krishnan, K. Derivation of Biomonitoring Equivalents for Di(2-Ethylhexyl)Phthalate (CAS no. 117-81-7). Regul. Toxicol. Pharmacol. 2009, 55, 249-258. https://doi.org/10.1016/j.yrtph.2009.09.001.
7. Schulz, C.; Wilhelm, M.; Heudorf, U.; Kolossa-Gehring, M. Update of the Reference and HBM Values Derived by the German Human Biomonitoring Commission (Reprinted from International Journal of Hygiene and Environmental Health, Vol 215, Pg 26, 2011). Int. J. Hyg. Environ. Health 2012, 215, 150-158. https://doi.org/10.1016/j.ijheh.2012.01.003.
8. Aylward, L.L.; Hays, S.M.; Gagne, M.; Krishnan, K. Derivation of Biomonitoring Equivalents for Di-n-Butyl Phthalate (DBP), Benzylbutyl Phthalate (BzBP), and Diethyl Phthalate (DEP). Regul. Toxicol. Pharmacol. 2009, 55, 259-267. https://doi.org/10.1016/j.yrtph.2009.09.003.
9. Hays, S.M.; Aylward, L.L.; Kirman, C.R.; Krishnan, K.; Nong, A. Biomonitoring Equivalents for Di-Isononyl Phthalate (DINP). Regulatory Toxicology and Pharmacology 2011, 60, 181-188. https://doi.org/10.1016/j.yrtph.2011.03.013.
10. Casas, M.; Valvi, D.; Luque, N.; Ballesteros-Gomez, A.; Carsin, A.; Fernandez, M.F.; Koch, H.M.; Mendez, M.A.; Sunyer, J.; Rubio, S. et al. Dietary and Sociodemographic Determinants of Bisphenol A Urine Concentrations in Pregnant Women and Children. Environ. Int. 2013, 56, 10-18. https://doi.org/10.1016/j.envint.2013.02.014.
11. Mustieles, V.; Ocon-Hernandez, O.; Minguez-Alarcon, L.; Davila-Arias, C.; Perez-Lobato, R.; Calvente, I.; Arrebola, J.P.; Vela-Soria, F.; Rubio, S.; Hauser, R. et al. Bisphenol A and Reproductive Hormones and Cortisol in Peripubertal Boys: The INMA-Granada Cohort. Sci. Total Environ. 2018, 618, 1046-1053. https://doi.org/10.1016/j.scitotenv.2017.09.093.
12. Cutanda, F.; Koch, H.M.; Esteban, M.; Sanchez, J.; Angerer, J.; Castano, A. Urinary Levels of Eight Phthalate Metabolites and Bisphenol A in Mother-Child Pairs from Two Spanish Locations. Int. J. Hyg. Environ. Health 2015, 218, 47-57. https://doi.org/10.1016/j.ijheh.2014.07.005.
13. Casas, L.; Fernandez, M.F.; Llop, S.; Guxens, M.; Ballester, F.; Olea, N.; Basterrechea Irurzun, M.; Santa Marina Rodriguez, L.; Riano, I.; Tardon, A. et al. Urinary Concentrations of Phthalates and Phenols in a Population of Spanish Pregnant Women and Children. Environ. Int. 2011, 37, 858-866. https://doi.org/10.1016/j.envint.2011.02.012.
